# Visuo-Motor Coordination Deficits and Motor Impairments in Parkinson's Disease

**DOI:** 10.1371/journal.pone.0003663

**Published:** 2008-11-06

**Authors:** Rivka Inzelberg, Edna Schechtman, Shraga Hocherman

**Affiliations:** 1 The Sagol Neuroscience Center and Department of Neurology Sheba Medical Center, Tel Hashomer, Israel; 2 Department of Industrial Engineering and Management, Ben Gurion University, Beer Sheva, Israel; 3 Department of Physiology, Faculty of Medicine, Technion, Haifa, Israel; Baylor College of Medicine, United States of America

## Abstract

**Background:**

Visuo-motor coordination (VMC) requires normal cognitive executive functionality, an ability to transform visual inputs into movement plans and motor-execution skills, all of which are known to be impaired in Parkinson's disease (PD). Not surprisingly, a VMC deficit in PD is well documented. Still, it is not known how this deficit relates to motor symptoms that are assessed routinely in the neurological clinic. Such relationship should reveal how particular motor dysfunctions combine with cognitive and sensory–motor impairments to produce a complex behavioral disability.

**Methods and Findings:**

Thirty nine early/moderate PD patients were routinely evaluated, including motor Unified Parkinson's Disease Rating Scale (UPDRS) based assessment, A VMC testing battery in which the subjects had to track a target moving on screen along 3 different paths, and to freely trace these paths followed. Detailed kinematic analysis of tracking/tracing performance was done. Statistical analysis of the correlations between measures depicting various aspects of VMC control and UPDRS items was performed. The VMC measures which correlated most strongly with clinical symptoms represent the ability to organize tracking movements and program their direction, rather than measures representing motor-execution skills of the hand. The strong correlations of these VMC measures with total UPDRS score were *weakened* when the UPDRS hand-motor part was considered specifically, and were insignificant in relation to tremor of the hand. In contrast, all correlations of VMC measures with the gait/posture part of the UPDRS were found to be strongest.

**Conclusions:**

Our apparently counterintuitive findings suggest that the VMC deficit pertains more strongly to a PD related change in cognitive-executive control, than to a reduction in motor capabilities. The recently demonstrated relationship between gait/posture impairment and a cognitive decline, as found in PD, concords with this suggestion and may explain the strong correlation between VMC dysfunction and gait/posture impairment. Accordingly, we propose that what appears to reflect a motor deficit in fact represents a multisystem failure, dominated by a cognitive decline.

## Introduction

It is long known that visuo-motor coordination (VMC) is deficient in patients with Parkinson's disease [1]–[7]. This deficit is found in early [8], [9], as well as in moderate [10] and advanced patients, and appears to pertain to a high level linkage between perception and action [11]. Early studies ascribed the deficit in VMC to a difficulty generating an inner model of the tracking task and in utilizing predictive planning in an “open loop” mode [1]–[3], but see [4]. Later studies have identified separate functional domains within which VMC is impaired, including reduced ability to assimilate the extent and speed of target movement during tracking [6], [9], and reduced ability to control the direction of hand movement during free tracing of a model path [9]–[11]. These deficits are not measured within the framework of a standard neurological examination. Yet, the correlations between them and other PD related dysfunctions may extend our understanding of the impairments afflicted by PD.

It is likely that basic motor disabilities such as bradykinesia, rigidity and tremor would interfere with tracking of a visually moving target and with tracing of a given geometrical shape. Yet, it is also possible that deficient motor planning, together with declining cognitive executive capabilities [8], [12], which drive performance, contribute to this failure. This problem can not be resolved by merely observing that VMC is already markedly impaired in patients with mild motor symptoms. A systematic analysis of the relationship between components of the motor part (part III) of the Unified Parkinson's Disease Rating Scale (UPDRS, [13]) and individual components of visuomotor performance is necessary in order to clarify this issue. If VMC dysfunction results only from low motor capabilities of the executing limb, strong correlations with limb items of the UPDRS should be expected. Alternatively, if the VMC deficit derives mainly from deficient visuo-motor planning and/or executive capabilities, such correlations would be low. On the other hand, correlations between UPDRS items unrelated to the performing limb, which are influenced by cognition, and VMC performance variables that relate to executive control may predominate. In the context of the latter possibility, recent findings show a significant interplay between cognition and posture/gait in PD patients [14]–[18]). Accordingly, a positive correlation between VMC measures that pertain to cognition and posture/gait would support this possibility.

Finally, assessment of VMC provides sensitive monitoring for the effects of drug treatment [19], [20] and may facilitate differential diagnosis of PD [21], [22]. Therefore, it is of practical interest, as well, to determine the relationship between the extent and nature of the VMC deficit and standard clinical measures of PD severity.

## Methods

### Subjects

Thirty-nine right-handed PD patients (19 males) at a Hoehn & Yahr stage I–III were studied. All patients were diagnosed by a movement disorders specialist (RI), using standard criteria [23]. The patients' mean age was 64.8±11.8 (SD) years and mean disease duration was 7.9±7.8 years. All subjects were right handed and were examined and tested during their “on” period, under their standard drug regimen. The experimental protocol was approved by the institutional review board of the Hillel Yaffe Medical Center, Hadera, Israel. All study participants signed a written informed consent form before entering this study.

### Clinical evaluation

All subjects underwent a standard neurological examination which included the motor part (part III) of the UPDRS. Next, a detailed instrumental VMC testing was performed by a technician blinded to the UPDRS scores.

### Visuo-Motor-Coordination (VMC) testing: Apparatus and procedure

**Figure 1:**
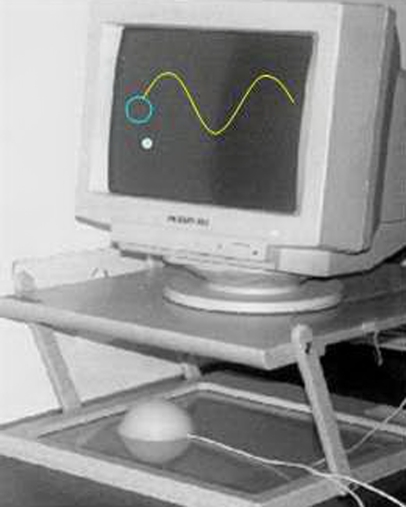
Apparatus for VMC testing. A digitizing tablet is enclosed in the bottom part, with a dome-shaped manipulandum resting over it. A sinusoidal path with a target at its left end and a subject-controlled cursor are shown on screen.

A computer based system is used for VMC testing (Figure 1). A digitizing tablet is placed horizontally below an elevated board, supporting a computer monitor. On top of the tablet lies a dome-shaped manipulandum which can slide freely over the tablet's surface. Both tablet and manipulandum are hidden from the subjects view by the overlying board. Movements of the manipulandum translate into movements of a point cursor on the computer screen. In addition to the cursor one out of three paths (see below) is displayed, together with a 1 cm target circle (in case of a tracking trial) or without it (in case of a tracing trial). Trajectory of the manipulandum is sampled at 1000 Hz, with a spatial resolution of 0.05 mm. Every 10^th^ sample is stored on disk for off line analysis. A one to one correspondence between movement of the manipulandum and movement of the screen cursor is preserved. VMC testing consists of ‘tracking’ trials and of ‘tracing’ trials.

Tracking trials: a path, having a total length of 32 centimeters, with either a sine wave, a square or a circular shape, is displayed on screen, together with a 1 cm/diameter target circle at one of its ends. The subject's task is to bring the cursor into the target and maintain the cursor within the target while it moves along the displayed path, from one end to the other. A constant target velocity of 18 mm/sec is applied to the square and circular paths. The same mean velocity is used for the sine-wave path but here target velocity varies from a minimum of 16 mm/sec at the peaks and troughs of the sine wave to 20 mm/sec along its middle sections. In case of a cursor exit from the target the target freezes in place (‘tracking interruption’) until the curser is reinserted into it.

Tracing trials: the subject's task is to move the cursor at his/her own pace along the displayed path (i.e., sinewave, square, circle) from one end to the other, with no restrictions on cursor velocity. The model path is displayed in white and cursor trajectory is shown in green.

All trials are performed alternatively with each hand (total of 12 trials). Tracking and tracing trials are interleaved systematically.

Several variables serve to assess the quality of performance during VMC testing. These variables are listed in Table 1. Data on tracking persistence, assimilation of target speed, and proximity to target center (top 4 rows of Table 1) are derived from the tracking trials. Proximity to the model path directional control and hand velocity are derived from both tracking and tracing trials. Each variable in Table 1 represents the average value for that measure in the relevant type of trial (tracking or tracing), with one hand (right or left), across all three path types (sine-wave, square, circle).

**Table 1:** Variables used for assessment of VMC performance.

VMC Variable	Abbreviation	Description
Percentage of lost tracking time	%Lost_Tm	Mean percentage of trial time that was spent with the cursor outside the target.
Frequency of Interruptions	Ints/Sec	Mean number of interruptions per second of tracking.
Mean Duration of Interruption	Int_Dur	Mean duration of a single tracking interruption, given in seconds.
Distance from target	Dist_T	Mean distance of the tracking cursor from target center, given in mm.
Distance from path	Dist_P	Mean separation between the model path and cursor trajectory, given in mm.
Directional tracing error	Dir_Er	Mean instantaneous component of the hand movement vector, perpendicular to the model path, given as % of the total movement vector.
Tracing duration	TTm	Mean time required for completion of a tracing trial.
Hand velocity	V	Mean hand movement velocity, given in mm/sec.

### Data analysis

The present study focused on the correlation between clinical measures of PD related disability, as expressed by the motor part of the UPDRS and visuo-motor coordination, as expressed by the VMC variables. Statistical analysis concerned the total score of the motor UPDRS, as well as the total scores of three specific groups of UPDRS items. One group pertained to upper limb function and included rigidity+finger taps+hand movements+rapid alternating movements (items 22+23+24+25 of the UPDRS). The second, one item group pertained specifically to upper limb rest tremor (item 20). The third group pertained to posture and gait and included posture+gait+postural stability (items 28+29+30).

Statistical analysis was performed in two steps. In the first step we estimated the correlations among all individual variables involved, namely: between variables derived from the motor UPDRS examination and those derived from the VMC. Spearman correlation was employed due to the non-normal distribution of the data (UPDRS measures are based on ranks). The second step was testing whether the UPDRS groups of items, as described above, had equal mean correlations with the VMC variables. In order to achieve this, we used the absolute values of the spearman correlation coefficients that were obtained in the first step as the dependent variable in a one way ANOVA with the UPDRS items groups as the fixed factor and the different VMC variables as blocks. Fisher's LSD procedure was then used in order to identify possible clustering of the items groups.

A third step of the analysis reviewed the internal relationships between the various VMC measures. As noted above, these measures pertain to cognitive-executive functionality (frequency of tracking interruptions), ability to transform visual spatial information into an appropriate command for hand-movement direction (directional error in tracing), ability to transform visual-movement information into an appropriate command for hand-movement direction (directional error in tracking), ability to use visual feedback in order to minimize deviations from the desired hand movement path (distance from path in tracing) and ability to exercise normal motor control of the hand (velocity of hand movement during tracing) [9], [10], [24]. These, and other multi factor measures (i.e. Total tracking time, Total tracing time, Distance from target center and more) do not change uniformly in PD [9], [10]. Therefore, it was important to determine their degree of independence before conclusions about their correlations with the UPDRS items groups could be drawn. The correlations between different VMC measures were determined by use of linear regression analysis.

## Results

**Figure 2:**
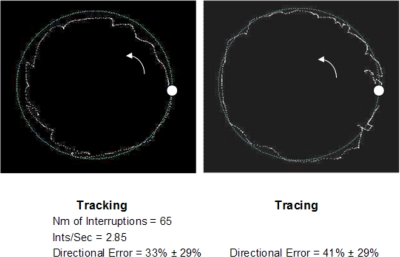
Tracking (left) and tracing (right) along a circular path, as performed by a single moderate PD patient. Each test started from the white dot and continued counter clockwise (indicated by the inserted arrow). The number of tracking interruptions, their rate and the mean±SD of the directional error for the above tests are shown. In elderly control subjects (n = 57) the mean values of these measures are: 7.0±3.7 (nm. of interruptions), 0.32±0.17 (Ints/Sec) and 24.8±1.3 (Dir_Er in tracing).

Mean age, disease duration, disease stage, UPDRS score and levodopa dosage of all subjects are reported in Table 2. All subjects were able to perform the entire battery of VMC tests. However, as previously reported [9], [10], typical PD related difficulties in test performance were noted in all cases, with the degree of deficit varying from one subject to another. Typical performance of one, moderate stage patient, in a single tracking task and in a single tracing task, along a circular path, is illustrated in figure 2. It can be seen that neither tracking nor tracing are smooth and that the measures depicting VMC performance errors have high values relative to mean normal control values that are taken from our previous database.

**Table 2:** Descriptive statistics of the 39 patients in the study group.

	Maximum	Minimum	Mean	S.D.
Age (years)	83.0	39.0	64.79	11.83
Duration (years)	38.0	0.6	7.89	7.75
H&Y Stage	3	1	2	1
UPDRS_R	41.0	.0	18.7	9.6
UPDRS_L	37.0	2.0	17.5	9.0
L-dopa (mg per day)	1000	0	319	468

Standard measures of VMC performance correlated moderately with the total UPDRS score. This includes the mean distance from target center (R = 0.36; p = 0.014), mean distance from the tracing path (R = 0.29; p = 0.041) and mean total tracing time (R = 0.37; p = 0.012). No significant correlation was found between the total UPDRS score and tracing speed (R = −0.13; n.s.). Stronger correlations were found with VMC measures specific to the present testing system. These include the frequency of tracking interruptions and the percentage of lost tracking-time (R = 0.65, p<1*10^−5^ for each). Both measures reflect the subject's ability to persist in tracking. The ability to perform the necessary visuo-motor transformations, too, as expressed by the directional tracing error [24] correlated well with the total UPDRS (R = 0.43, p = 0.004).

### Correlations between VMC and UPDRS items groups

The correlations between specific UPDRS items groups and the various VMC measures are shown in Table 3. It can be seen that these correlations are not of the same strength for all items groups, as described below.

**Table 3:** Correlation between clinical variable groups measures of PD manifestation and individual measures of VMC performance with each hand.

Spearman correlations between UPDRS Variable Groups and VMC variables							
		VMC test-Left hand			VMC test-Right hand		
		Hand	Rest tremor	Posture gait	Hand	Rest tremor	Posture gait
Ints/Sec	R	**0.49**	0.13	**0.71**	**0.50**	0.14	**0.69**
	P	0.0016	0.44	<.0001	0.0011	0.39	<.0001
%Lost_Tm	R	**0.51**	0.09	**0.72**	**0.47**	0.14	**0.77**
	P	0.0009	0.59	<.0001	0.0026	0.39	<.0001
Int_Dur	R	0.04	−0.01	0.18	0.22	0.03	**0.50**
	P	0.78	0.93	0.26	0.18	0.83	0.0013
Dist_T	R	**0.43**	0.10	**0.66**	0.27	0.09	**0.65**
	P	0.0064	0.54	<.0001	0.098	0.59	<.0001
Dist_P (k)	R	**0.49**	0.20	**0.69**	0.13	0.25	**0.52**
	P	0.0015	0.22	<.0001	0.42	0.13	0.0006
Dir_Er (k)	R	**0.42**	0.07	**0.72**	**0.42**	0.18	**0.71**
	P	0.008	0.65	<.0001	0.0077	0.27	<.0001
V (k)	R	**−0.41**	−0.30	**−0.32**	**−0.49**	−0.12	**−0.51**
	P	0.0087	0.06	0.0495	0.0014	0.46	0.0008
Dist_P (c)	R	**0.39**	0.05	**0.58**	0.31	0.04	**0.61**
	P	0.015	0.76	0.0001	0.053	0.81	<.0001
Dir_Er (c)	R	**0.47**	0.06	**0.47**	**0.53**	0.08	**0.61**
	P	0.0023	0.70	0.0027	0.0005	0.62	<.0001
V (c)	R	−0.29	−0.22	−0.12	−0.26	−0.08	−0.07
	P	0.069	0.18	0.45	0.11	0.64	0.68

R: Spearman correlation coefficient. P: Significance of the correlation coefficient. Significant R values (p≤0.05) are emphasized in bold font. The letters c and k that are added in parentheses to some variable names indicate whether the measure pertains to tracing (c) or to tracking (k).

The Posture & Gait items group correlated best with most VMC measures (Table 3). The correlation strength of all individual VMC measures with this items group, for right hand testing are, in a descending order: % lost tracking time (R = 0.77), directional error during tracking (R = 0.71), frequency of tracking Interruptions (R = 0.69), distance from target center (R = 0.65), directional error during tracing (R = 0.61), deviation from path during tracing (R = 0.61), deviation from path during tracking (R = 0.52), tracking speed (R = −0.51) mean duration of a tracking interruption ( R = 0.50) and tracing velocity (R n.s.).

A similar, though not identical pattern of correlations, can be seen for left hand performance of the VMC.

A weaker correlation with all VMC measures was found for the upper limb group of UPDRS variables (Table 3). The VMC variables that correlated significantly with the right limb's clinical deficit were, in a descending order of correlation strength: Directional error during tracing (R = 0.53), frequency of tracking Interruptions (R = 0.50), tracking speed (R = −0.49), % lost tracking time (R = 0.47) and directional error during tracking (R = 0.42). All other VMC measures did not correlate significantly with the right upper limb UPDRS items group score. Similar tendencies were found for the left upper limb items group correlations, with an additional significant correlation of the distance from path during tracking (R = 0.39).

As for the relationship to tremor of the hand, no VMC measure correlated significantly with this single variable of the right or left upper limb clinical status (p>0.1).

### Clustering of items groups

One way ANOVA with blocks showed that the mean correlation strengths for the 3 UPDRS items groups with the VMC measures were not all equal for the right as well as for the left hand (p<0.0001). Therefore, Fisher's LSD procedure was used in order to identify possible clustering of the items groups. This analysis showed that for the right hand the pooled VMC variables correlated most strongly with the posture/gait items group (average r = 0.619) and that this average correlation was significantly different (alpha = 0.05) than that formed with the upper limb group (average r = 0.377). The weakest and significantly lowest average correlation strength occurred with the upper limb's rest tremor (average r = 0.136) which constituted a separate group.

For the left hand, too, the highest correlation was found with the posture/gait items group (average r = 0.553), then, with a significantly lower correlation values (p<0.05) with the upper limb group (average r = 0.399). Finally, the weakest correlation was found for the upper limb's rest tremor item average (r = 0.118).

**Figure 3:**
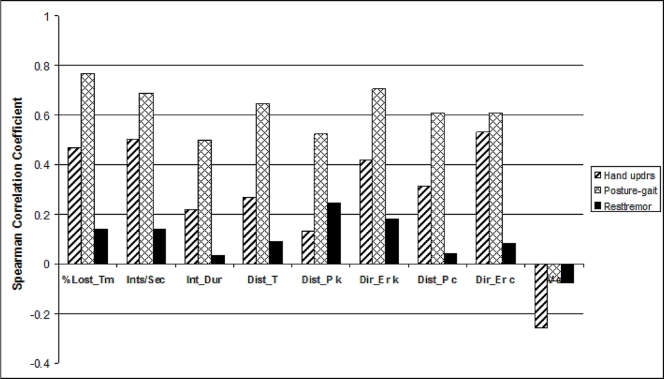
The relationship between all VMC measures (abscissa) and the various UPDRS items groups. Ordinate: Spearman correlation coefficient value. Insert: Designation of the different UPDRS items groups.

The above findings are summarized graphically in Figure 3, which shows the mean correlation strength of each individual VMC measure with the three UPDRS item groups. It can be seen that the only VMC measure with which the hand items group had the highest correlation was the velocity of hand movement during tracing. All other measures correlated most strongly with the posture/gait items group.

### Correlations between separate VMC measures

**Figure 4:**
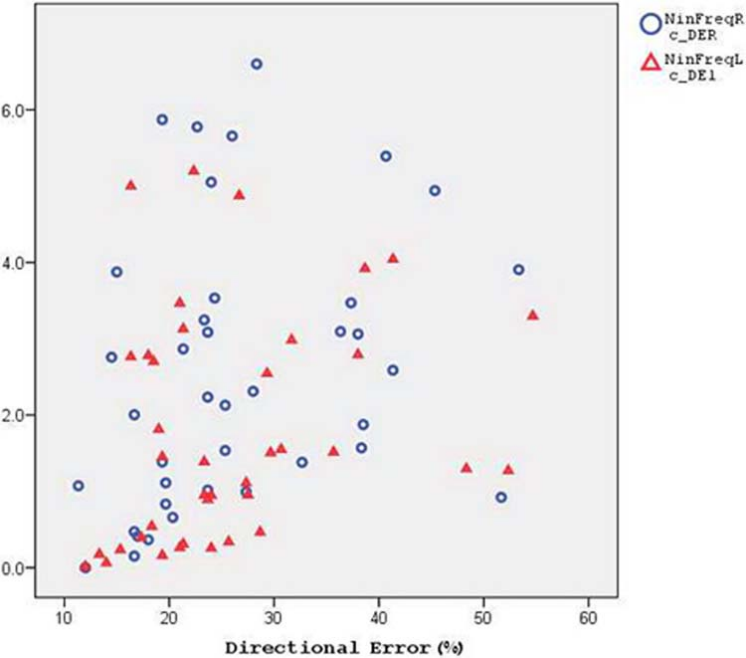
Relationship between the frequency of tracking interruptions and the directional error in tracing. The results of each patient are shown for the right hand (blue circles) and for the left hand (red triangles) performance of the VMC test battery. Abscissa: mean directional error in tracing. Ordinate: mean frequency of tracking interruptions. Abbreviations: NinFreqR – Frequency of tracking interruptions with the right hand. C_DER – Directional error in tracing with the right hand. Same abbreviations when terminated with “L” apply to performance with the left hand.

As explained above, different measures of VMC pertain to different functional domains. Most importantly, the frequency of tracking interruptions relates most strongly to cognitive executive functionality which underlies the ability to persist in tracking [10], [25], while the directional error in tracing relates most strongly to production of appropriate visuo-motor transformations [24]. Whether these variables are correlated determines how their relationships to the UPDRS item groups can be interpreted. Linear regression analysis revealed no significant correlation between these VMC measures for either the right or the left hand. This is shown in Figure 4, which presents a scatter plot of the frequency of tracking interruptions (Y axis) as a function of the tracing directional error (X axis). The measure of distance from the model path, too, did not correlate significantly with the frequency of tracking interruptions for either the right or the left hand. Thus, independent effects of PD on cognitive/executive functionality and on the ability to perform visuo-motor transformations can be assumed.

Of interest is also the between hands consistency of the above measures. A strong, highly significant correlation between the frequency of tracking interruptions with the right and left hand was found (Pearson correlation, r =  0.834, p<10^−6^). Similar findings were obtained with the directional error (Pearson correlation, r = 0.856, p<10^−6^).

## Discussion

The present study examines the relationship between visuo-motor coordination and clinical motor impairments in PD. This is done by examining the correlations between the overall, as well as particular functional groups of motor UPDRS items, with different measures of VMC compatibility. Surprisingly, we find that almost all measures of VMC compatibility relate more strongly to the UPDRS posture-gait items than to items directly reflecting a motor disability of the performing limb. To understand these findings it is first necessary to consider the skills underlying VMC, as discussed below.

Three functional domains of skills are involved in VMC performance. First, proper executive functionality is needed in order to organize the sequence of movements which allow the hand to remain within the boundaries of the moving target [25]. Second, successful transformation of visual information about target trajectory, target position and instantaneous position of the tracking cursor, into movement plans, is essential for performance [24]. Third, hand motor capabilities must meet the demands of the pending movements. Early studies suggested an effect of bradykinesia, relating the VMC deficit to limited manual motor capabilities of PD. However, it was soon discovered that deficits in the internal organization of tracking movements [3], [6], [10], reduced ability to act in a feed-forward mode and problems with internal representation of the target path [1]–[3], [26] play a more important role in hampering VMC. Accordingly, the relatively low correlation between VMC performance and the UPDRS hand items group is *not* the surprising finding of the present study. Furthermore, it has already been shown that tremor by itself does not lead to a decline in VMC [21], [22]. Thus, the finding in need of an explanation is the high correlation of most VMC variables with the posture/gait items group.

As shown before, the quality of tracking is influenced mainly by a cognitive-executive disability that can be linked to frontal cortical dysfunction in PD [25]. In addition, the basal ganglia (BG) are known to exert direct control over the pedunculo-pontine nuclei and through this pathway be involved in the selection of appropriate context relevant actions, pertaining to the coordination of gait [27]. Within this framework the BG are believed to be involved in on/off switching of different gait components in a way that ensures matching of the overall behavioral outcome to the organism's intention [27]. This role is very similar to the organizing role which must be played during tracking [3], [6], [10], [24], [25], as tracking itself consists of performing a series of small movement segments. If the BG mechanism that is involved in organizing the sequence of actions during normal stepping is faulty, it would be plausible to extrapolate that other sequencing-dependent actions, such as tracking, would be impaired. Indeed, it was found that PD patients could move their hand at the required tracking velocity [10] but were impaired in driving the tracking process consistently enough [9], [10], [25]. Thus, the high correlation between the posture/gait items and the VMC variables which represent the continuous flow of tracking movements (number of tracking interruptions and all associated variables) validate the above expectation.

Stance too is a dynamic process that requires continuing adjustments. In a study of postural responses to translational stance perturbations Horak et al [28] found that EMG activation of the patients' leg muscles was fragmented into multiple bursts, indicating inappropriate activation of the corrective stance synergy. Impaired postural stability in PD, therefore, did not appear to result from lack of motor tools or an inability to express them. Rather, it revealed a deficit in the ability to organize these tools into a fast enough effective response [28]. Obviously, this is the same case as with impaired manual tracking.

Finally, if gait and visuo-motor coordination share common executive resources and these resources diminish, as in PD, interference between them may become detrimental. This was demonstrated by studying the effect of performing an eye-hand coordination task on the gait of PD patients and elderly controls [29]. Subjects walked while carrying a tray with/without four glasses on it. Stabilizing the glasses on the tray caused a significantly greater deterioration of gait in the patients than in the controls.

According to current belief, the deficient ability to persist in tracking is part of a PD related frontal cortical dysfunction. However, the presently revealed correlation with gait/posture dysfunction is beyond this interpretation and suggests a substantial involvement of a brainstem component as well. Full details about the relationship between the cortical and subcortical circuits that are involved in executive control must await further research on this subject.

In the present study strong correlations were also found between the UPDRS gait/posture items and the measures of directional control in VMC. VMC is known to be organized by parietal-premotor circuits [30], [31]. The activity in this circuit, too, is modified in PD due to abnormal BG influences [32], resulting in lower precision of the tracking/tracing movements. In fact, this can be observed during VMC testing when occasional movements that are intended to bring the cursor closer to the target or to the traced path are misdirected and cause an increase in the tracking/tracing error. Such events appear as if reflecting inability of PD patients to construct a mental image of their tracking task [1.2] but may actually indicate a faulty utilization of this mental model.

Again, recent findings show that parallel subcortical circuits are also involved in VMC [31]. The main one includes the pontine nuclei, which receive massive inputs from the parietal visual areas, transmitting them to the cerebellum. The properties of pontine neuronal receptive fields are consistent with their role in providing a major link within the circuit that controls the visual guidance of movement [31]. Furthermore, evidence to the functional importance of this circuit exists [31]. Thus, although the relative contribution of the cortical and subcortical circuits in VMC is not fully understood, it is clear that a pathology that would disrupt pontine neuronal activity would cause some degree of visuo-motor dysfunction.

Apparently, different pontine neuronal groups may underlay the executive process of sequencing movement segments and of computing their direction, in parallel to the separate cortical fields that manage executive functionality, as opposed to visuo-motor transformations. Our results, showing no correlation between the frequency of tracking interruptions and the directional error in tracing are congruent with this dichotomy. Therefore we must conclude that directional control and process control are two separate dimensions of VMC, which correlate independently with the UPDRS gait/posture group of items.

In summary, the present findings show that the PD related impairment in VMC is multidimensional in functional terms and in brain systems terms. In functional terms it relates significantly to the motor impairment of the performing limb, but it relates more strongly to other clinical dimensions, such as gait and posture, whose common grounds with VMC are indirect and likely to involve cognitive/executive control. At the systems level we suggest that brainstem components that are influenced by PD play significant roles in what appears to be cortically controlled executive and computational skills. Thus, changes in VMC capabilities, as seen in PD patients, underscores the multi-dimensionality of basal ganglia involvement in cognitive, computational and motor aspects which underlay normal function.
